# Antioxidant and Anti-Osteoporosis Activities of Chemical Constituents of the Stems of *Zanthoxylum piperitum*

**DOI:** 10.3390/molecules23020457

**Published:** 2018-02-18

**Authors:** Seo Young Yang, Sang-Hyun Lee, Bui Huu Tai, Hae-Dong Jang, Young Ho Kim

**Affiliations:** 1 College of Pharmacy, Chungnam National University, Daejeon 34134, Korea; syyang@cnu.ac.kr; 2 Department of Food and Nutrition, Hannam University, Daejeon 34054, Korea; blackbean10@naver.com (S.-H.L.); haedong@hnu.ac.kr (H.-D.J.); 3 Institute of Marine Biochemistry (IMBC), Vietnam Academy of Science and Technology (VAST), Hanoi 10000, Vietnam; bhtaiich@gmail.com

**Keywords:** *Zanthoxylum piperitum*, Antioxidant, Anti-osteoporosis, ORAC, CUPRAC, TRAP

## Abstract

Two new lignans, zanthoxyloside C (**1**) and zanthoxyloside D (**2**), together with nine known compounds comprising lignans (**3**–**5**), flavonoids (**6**–**8**), and phenolics (**9**–**11**), were isolated from the methanol extract of the stems of *Zanthoxylum piperitum*. All isolates were evaluated for their antioxidant and anti-osteoporotic activities using oxygen radical absorbance capacity (ORAC), cupric reducing antioxidant capacity (CUPRAC), and tartrate-resistant acid phosphatase (TRAP) assays. Compounds **7**–**10** showed peroxyl radical-scavenging capacities and **4**, **6**–**7**, and **9** showed reducing capacities. Moreover, compounds **3**, **6**–**9**, and **11** significantly suppressed TRAP activities. These results indicated that the stems of *Z. piperitum* could be an excellent source for natural antioxidant and anti-osteoporosis.

## 1. Introduction

Osteoporosis, one of the metabolic diseases of the bones, occurs when the balance between bone resorption and bone formation is lost. To maintain bone mass and skeletal homeostasis, the dynamic process of resorption and formation continues in the bone tissues. Two types of bone cells, osteoclasts and osteoblasts perform specific functions in bone remodeling. Osteoclasts absorb bone, while osteoblasts synthesize and fill bone matrix; bone mass depends on the reciprocal function of these cells. A typical adult always maintains a balance between the amount of bone resorption and bone formation. However, once osteoporosis develops, due to aging, hormone abnormality, or lack of exercise, one’s quality of life degrades as a result of severe pain and limited mobility [1,2,3,4,5].

Recently, there has been a growing interest in the relationship between osteoporosis and oxidative stress. Clinical studies have shown that there is a significant correlation between increased oxidative stress and decreased bone mineral density. The antioxidant levels in the blood of osteoporotic women turned out to be low, but their bone mineral density increased by taking antioxidant vitamins. It has been revealed in in vitro studies, as well, that reactive oxygen species (ROS) increase the activity of osteoclasts and depress the metabolism of osteoblasts. The inhibition of the metabolism of osteoblasts owing to oxidative stress can be mitigated by the medication of antioxidants [6,7,8]. Therefore, if natural products without side effects on the human body were able to indirectly increase the intravital antioxidant defense system and directly eliminate excessive ROS, natural antioxidants could be applied as a functional material to prevent diseases caused by oxidative stress.

*Zanthoxylum piperitum* DC, widely distributed in South-East Asia, is an aromatic shrub belonging to the Rutaceae family. The fruits of *Z. piperitum* have been used as traditional herbal medicine as well as as a condiment. Most previous studies of *Z. piperitum* have focused on the fruits and leaves [9,10,11,12]. Therefore, there is a lack of information on chemical constituents of *Z. piperitum* stems and their biological activities. As a part of our ongoing research into the bioactivity of natural products, eleven secondary metabolites were isolated from stems of *Z. piperitum*. Moreover, antioxidant and anti-osteoporosis activities of these compounds were evaluated.

## 2. Results and Discussion

**Figure 1:**
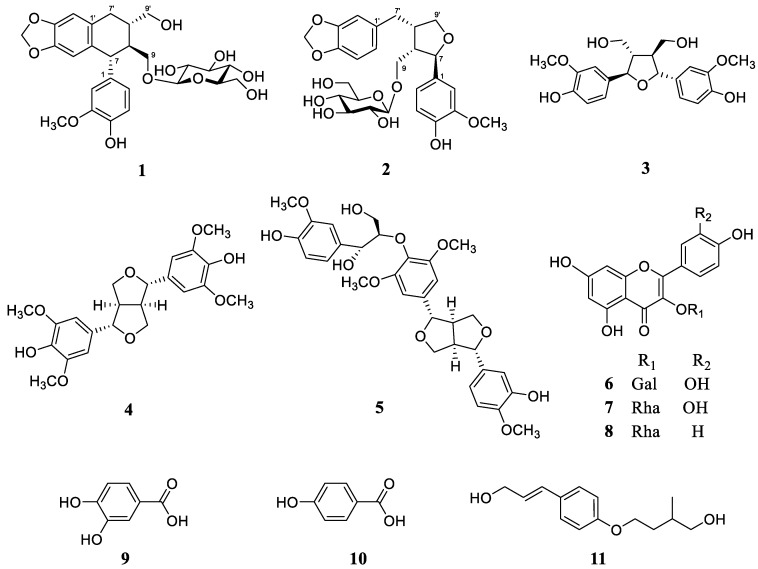
Structures of compounds **1**–**11** from *Z. piperitum* stems.

The phytochemical investigation of *Z. piperitum* stems resulted in the isolation of eleven compounds, including two new lignans, zanthoxyloside C (**1**) and zanthoxyloside D (**2**). The nine known compounds were determined to be (+)-neo-olivil (**3**) [13], (+)-syringaresinol (**4**) [14], hedyotol D (**5**) [15], hyperin (**6**) [16], quercitrin (**7**) [17], and kaempferol 3-*O*-rhamnoside (**8**) [18], protocatechuic acid (**9**) [19], 4-hydroxybenzoic acid (**10**) [20], and ailanthoidiol (**11**) [21] by comparing their NMR spectroscopic data with those of the published data (Figure 1).

**Figure 2:**
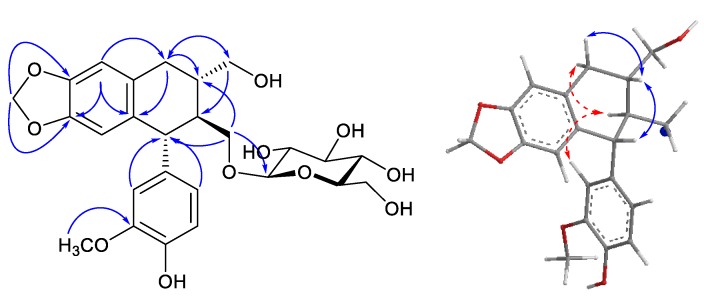
Key HMBC and NOESY correlations of compound **1**.

Compound **1** was obtained as a pale yellow amorphous powder. The HR-ESI-MS spectrum of compound **1** contained quasi-molecular ion peaks at *m*/*z* 543.1864 [M + Na]^+^ (Cald for C_26_H_32_NaO_11_, 543.1837), indicating its molecular formula to be C_26_H_32_O_11_. The ^1^H-NMR spectrum of **1** showed signals characteristic for a 1,3,4-trisubstituted benzene ring (δ_H_ 6.55 (1H, dd, *J* = 1.8, 7.8 Hz, H-6), 6.60 (1H, d, *J* = 1.8 Hz, H-2), and 6.65 (1H, d, *J* = 7.8 Hz, H-5)) and a 1′,2′,4′,5′-tetrasubstituted benzene ring (δ_H_ 6.04 (1H, s, H-3′) and 6.48 (1H, s, H-6′)). The signals of a methylenedioxy group and a methoxy group were observed at δ_H_ 5.70 (2H, s) and 3.70 (3H, s), respectively. Additionally, the signal of one anomeric proton at δ_H_ 3.96 (1H, d, *J* = 7.3 Hz) indicated the presence of one sugar unit in the structure of **1**. The ^13^C-NMR (Table 1) and DEPT-135 spectroscopic data indicated signals of 26 carbons. In addition to signals of a dioxygen-bearing methylene, a methoxy group, and a hexose sugar moiety, the remaining 18 carbon signals were assigned to two C6-C3 units. The HMBC spectrum revealed significant correlations between the proton signal at δ_H_ 6.48 (H-6′) and the carbon signals at δ_C_ 34.2 (C-7′), 134.5 (C-2′), 147.2 (C-5′), 147.3 (C-4′); between the proton signal at δ_H_ 2.76 (H-7′a) and the carbon signals at δ_C_ 41.0 (C-8′), 65.5 (C-9′), 131.4 (C-1′), 134.5 (C-2′); between the proton signal at δ_H_ 3.59 (H-9′b) and the carbon signals at δ_C_ 34.2 (C-7′), 45.5 (C-8); between the proton signal at δ_H_ 3.54 (H-9) and the carbon signals at δ_C_ 41.0 (C-8′), 49.7 (C-7), 104.1 (C-1″); and between the proton signal at δ_H_ 5.70 (-OCH_2_O-) and the carbon signals at δ_C_ 147.2 (C-5′), 147.3 (C-4′), which led to confirmation of the establishment of a tetralin glycoside moiety. Correlations between the proton signal at δ_H_ 6.60 (H-2) and the carbon signals at δ_C_ 49.7 (C-7), 123.5 (C-6), 138.6 (C-1), 146.24 (C-4), 149.2 (C-3); and between the methoxy proton signal at δ_H_ 3.70 and the carbon signal at δ_C_ 149.2 (C-3) in the HMBC spectra led to the confirmation of a hydroxyl and a methoxyl group at C-4 and C-3, respectively. Correlations between the proton signals at δ_H_ 6.60 (H-2), 6.55 (H-6) and the carbon signal at δ_C_ 49.7 (C-7) further indicated that two phenyl groups connected together at C-7 to form an aryltetralin lignan. The glucosyl linkage was confirmed by HMBC correlation between an anomeric proton at δ_H_ 3.96 (H-1″) and δ_C_ 70.8 (C-9). The coupling constant of the anomeric proton was 7.3 Hz in doublet multiplicity in the ^1^H-NMR spectrum, which confirmed the β-configuration of glucopyranoside. The absolute configurations were determined by examination of the circular dichroism (CD) spectrum. The CD spectrum of **1** showed a positive and a negative Cotton effect at 294 nm (+10.84) and 277 nm (−8.07), respectively, indicating “*R*” configuration of C-7, which was well characterized for 7(*R*)-aryltetralin lignans [22,23]. Next, NOE correlations of H-8 (δ_H_ 1.87) with both H-2 (δ_H_ 6.60) and H-6 (δ_H_ 6.55) indicated *S* configuration at C-8. Due to the close chemical shifts of H_2_-9 and H_2_-9′, the absolute configuration at C-8′ was determined by interpretation of ^13^C- and ^1^H-NMR spectra. The similar chemical shifts of C-7′ (δ_C_ 34.2), C-8 (δ_C_ 45.5), C-8′ (δ_C_ 41.0), and C-9′ (δ_C_ 65.5) were compared with those of (-)-isolariciresinol 3α-*O*-β-d-glucopyranoside (C-7′ (δ_C_ 33.6), C-8 (δ_C_ 45.4), C-8′ (δ_C_ 41.1), and C-9′ (δ_C_ 65.5)) [24], suggesting the same configuration “*R*” at C-8′. Additionally, the “*R*” configuration at C-8′ was in good agreement with the trans-axial coupling constant of H-8′ (δ_H_ 1.87) and H-7′ax (δ_H_ 2.76, dd, *J* = 16.1, 10.1 Hz) (Figure 2). Consequently, the structure of compound **1** was determined to be (7*R*,8*S*,8′*R*)-3-methoxy-4,9,9′-trihydroxy-3′,4′-methylendioxy aryltetralin lignan 9-*O-*β-d-glucopyranoside, and named zanthoxyloside C. 

**Figure 3:**
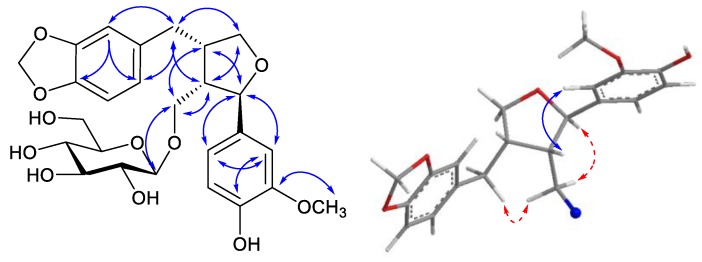
Key HMBC and NOESY correlations of compound **2**.

Compound **2** was obtained as a white amorphous powder. The HR-ESI-MS spectrum of compound **2** contained quasi-molecular ion peaks at *m*/*z* 543.1873 [M + Na]^+^ (Cald for C_26_H_32_NaO_11_, 543.1837), indicating its molecular formula to be C_26_H_32_O_11_. The ^1^H-NMR spectrum of **2** showed two 1,3,4-trisubstituted benzene ring spin systems (δ_H_ 6.71 (1H, dd, *J* = 2.1, 8.2 Hz, H-6), 6.83 (1H, d, *J* = 2.1 Hz, H-2), and 6.66 (1H, d, *J* = 8.2 Hz, H-5)) and (δ_H_ 6.59 (1H, dd, *J* = 8.2, 1.3 Hz, H-2′) , 6.63 (1H, d, *J* = 8.2 Hz, H-3′) and 6.64 (1H, dd, *J* = 8.2, 1.3 Hz, H-6′)). The signal of a methylene dioxy proton at δ_H_ 5.80 (2H, s) and a methoxy group at δ_H_ 3.76 (3H, s) were also observed. The signal of one anomeric proton at δ_H_ 4.20 (1H, d, *J* = 8.2 Hz) indicated the presence of one sugar unit in the structure of **2**. The ^13^C-NMR (Table 1) and DEPT-135 spectroscopic data of **2** also indicated signals of 26 carbons, which was in agreement with the structure of lignan glycoside as compound **1**. However, the downfield shift of C-7 (δ_C_ 84.3) and C-9′ (δ_C_ 73.7) suggested that compound **2** belonged the tetrahydrofuran lignans. The HMBC spectrum revealed significant correlations between the proton signal at δ_H_ 6.65 (H-6′) and the carbon signals at δ_C_ 33.9 (C-7′), 122.7 (C-2′), 136.1 (C-1′), 147.3 (C-4′); between the proton signal at δ_H_ 2.91 (H-7′a) and the carbon signals at δ_C_ 44.2 (C-8′), 51.8 (C-8), 73.7 (C-9′), 110.1 (C-6′), 122.7 (C-2′), 136.1 (C-1′); between the proton signal at δ_H_ 2.63 (H-8′) and the carbon signals at δ_C_ 51.8 (C-8), 68.5 (C-9), 73.7 (C-9′), 84.3 (C-7), 136.1 (C-1′); and between the proton signal at δ_H_ 2.41 (H-8) and the carbon signals at δ_C_ 33.9 (C-7′), 44.2 (C-8′), 68.5 (C-9), 73.7 (C-9′), 84.3 (C-7), which led to the establishment of a linkage moiety between methylenedioxyphenyl and tetrahydrofuran. Correlations between the proton signal at δ_H_ 6.83 (H-2) and the carbon signals at δ_C_ 84.3 (C-7), 119.9 (C-6), 135.6 (C-1), 147.1 (C-4), 149.2 (C-3); and between the methoxy proton signal at δ_H_ 3.76 and the carbon signal at δ_C_ 149.2 (C-3) in HMBC led to the establishment of a hydroxyl and methoxy groups at C-4 and C-3, respectively. Correlations between the proton signals at δ_H_ 6.83 (H-2), 6.70 (H-6) and the carbon signal at δ_C_ 84.3 (C-7) revealed a second benzene ring moiety connected to the tetrahydrofuran ring at C-7. The glucosyl linkage was confirmed by the HMBC correlation between an anomeric proton at δ_H_ 4.20 (H-1″) with δ_C_ 68.53 (C-8). The coupling constant of the anomeric proton was 8.2 Hz in doublet multiplicity in the ^1^H-NMR spectrum, which confirmed the β-configuration of glucopyranoside. Finally, the absolute configurations were determined by examinations of the CD spectrum and NOE correlation. The NOE correlations, including proton H-8 (δ_H_ 2.41) with both protons H-2 (δ_H_ 6.83) and H-6 (δ_H_ 6.71), proton H-7 (δ_H_ 4.73) with H-9 (δ_H_ 3.50), proton H-9 (δ_H_ 3.50) with H-7′ (δ_H_ 2.91), were clearly observed in the NOESY spectrum of **2**, which confirmed their close proximity, as shown in Figure 3. In addition, the CD spectrum of **2** showed the opposite trend of Cotton effects (positive effects at 241 nm (+0.24) and 289 nm (+0.13)) in comparison with those of (+)-(7*S*,8*R*,8′*R*)-lariciresinol (negative effects at 244 nm (−0.42) and 290 nm (−0.26)) _ENREF_4 [25], which indicated 7*R*,8*S*,8′*S* configurations of compound **2**. Thus, the structure of compound **2** was determined to be (7*R*,8*S*,8′*S*)-3-methoxy-4,9-dihydroxy-3′,4′-methylendioxy-7,9′-epoxylignan 9-*O*-β-d-glucopyranoside, and named zanthoxyloside D (see Supplementary Materials).

The antioxidant activities of the isolated compounds **1**–**11** were evaluated with respect to their peroxyl radical-scavenging and reducing capacity. Table 2 shows the scavenging activities of compounds **1**–**11** on peroxyl radicals, which were generated from 2,2′-azobis(2-amidinopropane) dihydrochloride (AAPH) in the oxygen radical absorbance capacity (ORAC) assay. All isolated compounds showed significant peroxyl radical-scavenging activities, with values of 5.91 ± 0.11 to 26.91 ± 1.05 μM at a concentration of 10 μM. The ability of compounds **1**–**11** to stimulate the reduction of copper ions (Cu^2+^ to Cu^+^) by donating electrons was investigated to determine whether their peroxyl radical-scavenging capacities, with the donation of hydrogen atoms, could be related to their reduction capacities. As shown in Table 2, compounds **1**–**9** showed significant reducing capacities, with values of 9.60 ± 0.26 to 33.04 ± 0.17 μM at a concentration of 10 μM. The rest of the compounds (**10** and **11**) showed weak activities. These results suggest that the peroxyl radical-scavenging and reducing capacity of all the tested compounds, due to transfer of hydrogen atoms and single electron, may be relevant to the hydroxyl groups of the benzene rings [26,27,28]. 

The anti-osteoporotic activities were investigated using TRAP assay on RAW 264.7 cells. The inhibitory effects of isolated compounds were tested based on the suppression of excessive bone resorption by osteoclasts. As shown in Table 3, compounds **3**, **6**–**9**, and **11** showed significant inhibitory activities, with values of 77.73 to 92.42% relative to the RANKL-treated control (100%).

## 3. Materials and Methods 

### 3.1. General Information

The NMR spectra were recorded using JEOL ECA 600 MHz, JEOL JNM-AL 400 MHz (Jeol, Tokyo, Japan), and Bruker FT 300 MHz (Bruker Biospin GmbH, Karlsruhe, Germany) spectrometer using TMS as an internal standard. Chemical shift (δ) is expressed in ppm with reference to the TMS signals. Low ESI-MS spectra were obtained on a Shimadzu LCMS-2010. High-resolution electrospray ionization mass spectra (HR-ESI-MS) were obtained using an Agilent 6530 Accurate-Mass Q-TOF LC/MS system. The CD spectra were recorded using Jasco J-815 (150-L) (JASCO Crop., Tokyo, Japan). The UV spectra were recorded using UVmini-1240 (Shimadzu, Kyoto, Japan). GC was carried out on a Shimadzu-2010 (Shimadzu, Kyoto, Japan) spectrometer: detector, FID; detection temperature, 300 °C; column, SPB-1 (0.25mm i.d. × 30 m); column temperature, 230 °C; carrier gas, He (2 mL/min) injection temperature, 250 °C; injection volume, 0.5 μL. Column chromatography was performed using silica gel (Kieselgel 60, 70–230 mesh and 230–400 mesh, Merck, Darmstadt, Germany) and C-18 resins (30–50 µm, Fuji Silysia Chemical Ltd., Kasugai, Japan).

### 3.2. Plant Material

Dried stems of *Z. piperitum* were purchased at Daekwang Farm, Busan, Korea in November 2012 and were taxonomically identified by one of the authors (Prof. Young Ho Kim). A voucher specimen (CNU12107) was deposited at the Herbarium of College of Pharmacy, Chungnam National University, Daejeon, Korea.

### 3.3. Extraction and Isolation

Dried stems of *Z. piperitum* DC. (3.0 kg) were extracted with methanol at room temperature three times. After removal of the solvent under reduced pressure, the crude extract (120.0 g) was dissolved in 4.0 L of H_2_O to form a suspension that was successively partitioned with *n*-hexane, CH_2_Cl_2_, EtOAc, and BuOH to give *n*-hexane (45.0 g), CH_2_Cl_2_ (29.0 g), EtOAc (2.5 g), and BuOH (28.0 g) extracts, respectively.

The CH_2_Cl_2_ extract was subjected to column chromatography using SiO_2_ (70,230 mesh), eluting with gradient solvent system of *n*-hexane/acetone (100/0–0:100; *v*/*v*, 1.5 L for each step) to give five fractions (D1–D6). Franction D2 (900.0 mg) was subjected to RP column, eluted with gradient solvent system of MeOH/H_2_O (3/7–4/1; *v*/*v*, 0.5 L for each step) to yield six sub-fractions (D2.1–D2.6). Fraction D2.3 (90.0 mg) was separated using silica gel column with *n*-hexane/CH_2_Cl_2_/MeOH (3/3/0.2, *v*/*v*/*v*) as eluent to afford compound **3** (5.0 mg). Fraction D2.5 (200.0 mg) was separated using sephadex LH-20 column with CH_2_Cl_2_/MeOH (2/1, *v*/*v*) as eluent to afford compound **4** (90.0 mg). Fraction D4 (3.1 g) was subjected to silica gel column chromatography, eluted with gradient solvent system of *n*-hexane/EtOAc/MeOH (14/2/1–7/2/1; *v*/*v*/*v*, 0.8 L for each step) to give five fractions (D4.1–D4.5). Fraction D4.3 (1.8 g) was separated by silica gel column eluting with n-hexane/EtOAc/acetone (10/4/1, *v*/*v*/*v*) to yield four smaller fractions (D4.3.1.1–D4.3.1.4). Fraction D4.3.1.2 (20.0 mg) was separated using RP column, eluted with MeOH/H_2_O (3/2, *v*/*v*) to yield compound **11** (4.0 mg). 

The EtOAc extract was subjected to column chromatography using sephadex LH-20, eluting with CH_2_Cl_2_/MeOH (1/1, *v*/*v*) to give five fractions (E1–E5). Fraction E2 (180.0 mg) was subjected to sephadex LH-20 column, eluted with MeOH/ H_2_O (1/1, *v*/*v*) to give four sub-fractions (E2.1–E2.4). Fraction E2.4 (20.0 mg) was subjected to RP column, eluted with gradient solvent system of MeOH/H_2_O (1/4–1/1; *v*/*v*, 0.4 L for each step) to yield compounds **9** (7.0 mg) and **10** (4.0 mg). Fraction E3 (900.0 mg) was subjected to RP column, eluted with a gradient solvent system of MeOH/H_2_O (1/4–0/1; *v*/*v*, 0.5 L for each step) to yield four sub-fractions (E3.1–E3.4). Fraction E3.2 (800.0 mg) was separated using silica gel column with CHCl_3_/MeOH/H_2_O (5/1/0.1, 3/1/0.1, *v*/*v*/*v*) elution solvent to give compounds **6** (750.0 mg) and **8** (12.0 mg). Fraction E3.3 (90.0 mg) was separated using silica gel column with CHCl_3_/MeOH/H_2_O (4/1/0.1, *v*/*v*/*v*) as eluent to afford compound **7** (73.0 mg). Fraction E4 (230.0 mg) was separated by RP column eluting with a gradient solvent system of MeOH/H_2_O (1:4–0:1; *v*/*v*, 0.4 L for each step) to yield four smaller fractions (E4.1–E4.4). Repeated silica gel column chromatography of fraction E4.4 with CH_2_Cl_2_/MeOH (10/1, *v*/*v*) and further purified using sephadex LH-20 column with MeOH/H_2_O (1/1, *v*/*v*) to give compounds **5** (70.0 mg). Fraction E5 (500.0 mg) was separated using silica gel column with *n*-hexane/EtOAc/acetone (10/4/1, *v*/*v*/*v*) to give six fractions (E5.1–E5.6). Fraction E5.5.1 (13.0 mg) was separated by RP column eluting with MeOH/H_2_O (2/1, *v*/*v*) to yield compound **1** (4.0 mg). Fraction E5.5.2 (10.0 mg) was subjected to silica gel column, eluted with *n*-hexane/EtOAc/MeOH (1/1/0.3, *v*/*v*/*v*) to obtain compound **2** (4.0 mg).

### 3.4. Acid Hydrolysis and Sugar Identification

Compounds **1** and **2** (2 mg each) were heated in 3 mL 10% HCl (dioxane-H_2_O, 1:1) at 90 °C for 3 h. The residue was partitioned between EtOAc and H_2_O to give aglycone and sugar, respectively. The aqueous layer was evaporated until dry to yield a residue; this was dissolved in anhydrous pyridine (200 μL) and then mixed with a pyridine solution of 0.1 M l-cysteine methyl ester hydrochloride (200 μL). After warming to 60 °C for 1 h, trimethylsilylimidazole solution was added, and the reaction solution was warmed at 60 °C for 1 h. The mixture was evaporated in vacuo to yield a dried product, which was partitioned between *n*-hexane and H_2_O. The *n*-hexane layer was filtered and analyzed by gas chromatography. Retention times of the persilylated monosaccharide derivatives were as follows: D-glucose (t_R_, 14.11 min) was confirmed by comparison with those of authentic standards (Sigma-Aldrich, St. Louis, MO, USA).

### 3.5. Product Characterization

*Zanthoxyloside C* (**1**): Pale yellow amorphous powder; C_26_H_32_O_11_; ${\left[\alpha \right]}_{D}^{25}$: −30.7 (*c* 0.1, MeOH), UV (MeOH) *λ*_max_ (nm) (log *ε*): 287 (3.78) nm, IR (KBr) ν_max_: 3365, 2891, 1616, 1435, 1232, 1073, 1034 cm^−1^; (^1^H-NMR (CD_3_OD, 600 MHz) and ^13^C-NMR data (CD_3_OD, 150 MHz), see Table 1; HR-ESI-MS: *m*/*z* 543.1864 [M + Na]^+^ (Cald for C_26_H_32_NaO_11_, 543.1837).

*Zanthoxyloside D* (**2**): White amorphous powder; C_26_H_32_O_11_; ${\left[\alpha \right]}_{D}^{25}$: +52.7 (*c* 0.1, MeOH), UV (MeOH) *λ*_max_ (nm) (log *ε*): 285 (3.65) nm, IR (KBr) ν_max_: 3392, 2886, 1635, 1436, 1248, 1075, 1035 cm^−1^; (^1^H-NMR (CD_3_OD, 600 MHz) and ^13^C-NMR data (CD_3_OD, 150 MHz), see Table 1; HR-ESI-MS: *m*/*z* 543.1873 [M + Na]^+^ (Cald for C_26_H_32_NaO_11_, 543.1837).

### 3.6. Oxygen Radical Absorbance Capacity (ORAC) Assay

ORAC assay was carried out using a Tecan GENios multifunctional plate reader (Salzburg, Austria) with fluorescent filters (excitation wavelength: 485 nm, emission filter: 535 nm). In the final assay mixture, fluorescein (40 nM) was used as a target of free radical attack with AAPH (20 mM) as a peroxyl radical generator in the peroxyl radical-scavenging capacity assay. The analyzer was programmed to record fluorescein fluorescence every 2 min after AAPH had been added. All fluorescence measurements were expressed relative to the initial reading. Final values were calculated based on the difference in the area under the fluorescence decay curve between the blank and test sample. All data are expressed as net protection area (net area). Trolox (1 µM) was used as the positive control to scavenge peroxyl radicals [29].

### 3.7. Reducing Capacity (CUPRAC) Assay

The electron-donating capacities of isolated compounds (**1**–**11**) to reduce Cu^2+^ to Cu^+^ were assessed according to the method of Aruoma et al [30]. Forty microliters of different concentrations of compounds dissolved in ethanol were mixed with 160 µL of a mixture containing 0.5 mM CuCl_2_ and 0.75 mM neocuproine, a Cu^+^ specific chelator, in10 mM phosphate buffer. Absorbance was measured using a microplate reader at 454 nm for 1 h. Increased absorbance of the reaction mixture indicated greater reducing power.

### 3.8. Tartrate-Resistant Acid Phosphatase (TRAP) Assay

TRAP Staining. RAW 264.7 cells (macrophages (pre-osteoclasts) from BALB/c mouse) were seeded in 12-well plates (3 × 10^4^ cells/well) containing DMEM medium plus 10% FBS, and the medium was replaced with test samples in differentiation medium containing 50 ng/mL RANKL. The differentiation medium was changed every 2 days. After 5 days, the medium was removed, and the cell monolayer was gently washed twice using ice-cold PBS. The cells were fixed in 3.5% formaldehyde for 10 min and ethanol-acetone (1:1) for 1 min. Subsequently, the dried cells were incubated in 50 mM citrate buffer (pH 4.5) containing 10 mM sodium tartrate and 6 mM PNPP. After 1 h incubation, the reaction mixtures were transferred to new well plates containing an equal volume of 0.1 N NaOH. Absorbance was measured at 405 nm using an enzyme-linked immunoassay reader, and TRAP activity was expressed as the percent of the untreated control [31].

### 3.9. Statistical Analysis

All data represent the mean ± S.D. of at least three independent experiments performed in triplicates. Statistical significance is determined by one-way ANOVA followed by Dunnett’s multiple comparison test, *p* < 0.05, using the SPSS 21 (IBM Crop., Armonk, NY, USA) program.

## 4. Conclusions

This study confirmed that the phenolic constituents of *Z. piperitum* stems have potentialities for antioxidant and anti-osteoporosis activities. When comparing the results of two activities, there was no significant correlation between antioxidant and anti-osteoporotic activities. Therefore, further study may be required to determine whether the significant anti-osteoporotic activities of compounds **3**, **6**–**9**, and **11** are indirectly related to the antioxidant activity.

## Figures and Tables

**Table 1:** ^1^H- and ^13^C-NMR spectroscopic data of compounds **1** and **2** in CD_3_OD.

Position	1		2	
	δ_C_^a^	δ_H_^b^ (mult., *J* in Hz)	δ_C_^a^	δ_H_^b^ (mult., *J* in Hz)
1	138.6	-	135.6	-
2	114.0	6.60 (d, 1.8)	110.7	6.83 (d, 2.1)
3	149.2	-	148.2	-
4	146.2	-	147.1	-
5	116.2	6.65 (d, 7.8)	116.0	6.66 (d, 8.2)
6	123.5	6.55 (dd, 7.8, 1.8)	119.9	6.71 (dd, 8.2, 2.1)
7	49.7	3.68 *	84.3	4.73 (d, 6.9)
8	45.5	1.87 *	51.8	2.41 (q, 6.8)
9	70.8	3.54 (dd. 6.2, 10.3)	68.5	3.50 (dd, 6.2, 10.3)
		3.69 (dd, 6.2, 10.3)		4.11 (dd, 6.2, 10.3)
1′	131.4	-	136.1	-
2′	134.5	-	122.7	6.59 (dd, 8.2, 1.3)
3′	110.5	6.04 (s)	109.1	6.63 (d, 8.2)
4′	147.3	-	147.3	-
5′	147.2	-	149.2	-
6′	108.9	6.48 (s)	110.1	6.64 (dd, 8.2, 1.3)
7′	34.2	2.76 (dd, 10.1, 16.1)	33.9	2.91 (dd, 3.1, 12.4)
		2.63 (dd, 2.8, 16.1)		2.45 (dd, 3.1, 12.4)
8′	41.0	1.87 *	44.2	2.63 (m)
9′	65.5	3.69 *	73.7	3.87 (dd, 6.2, 8.2)
		3.59 *		3.63 (dd, 6.2, 8.2)
3-OCH_3_	56.6	3.70 *	56.4	3.76 (s)
-OCH_2_O-	101.9	5.70 (s)	102.2	5.80 (s)
Glc-1	104.1	3.96 (d, 7.3)	104.7	4.20 (d, 7.6)
2	75.2	3.07 (m)	75.2	3.15 (dd, 9.1, 7.8)
3	78.3	3.19 (m)	78.3	3.21 (m)
4	71.5	3.20 (m)	71.7	3.28 (m)
5	78.0	2.99 (m)	78.0	3.34 (m)
6	62.6	3.56 (m)	62.9	3.61 (m)
		3.70 (m)		3.84 (m)

* Overlapped signals; assignments were done by HMQC, HMBC, and NOESY experiments. ^a^ Measured at 600 MHz. ^b^ Measured at 150 MHz.

**Table 2:** The antioxidant activities of compounds isolated from the stems of *Z. piperitum*.

Compound (10 μM)	Peroxyl Radical-scavenging Capacity (TE, μM) ^a^	Reducing Capacity (Copper(I) Ions, μM)
**1**	13.26 ± 0.33	9.60 ± 0.26
**2**	15.79 ± 0.64	10.69 ± 0.11
**3**	14.47 ± 0.98	14.51 ± 0.17
**4**	12.98 ± 0.30	20.42 ± 0.55
**5**	15.81 ± 0.33	16.39 ± 0.36
**6**	19.09 ± 0.09	25.66 ± 0.32
**7**	22.12 ± 0.76	27.97 ± 0.34
**8**	22.89 ± 0.87	10.19 ± 0.11
**9**	26.91 ± 1.05	33.04 ± 0.17
**10**	20.53 ± 0.89	0.04 ± 0.06
**11**	5.91 ± 0.11	0.08 ± 0.06

All data are expressed as the mean ± standard deviation of three individual experiments. ^a^ Values are expressed as μM of Trolox equivalents (TE), one ORAC unit is equivalent to the net protection area provided by 1 μM of Trolox.

**Table 3:** Inhibitory effects of the isolated compounds on RANKL-induced osteoclast differentiation. ^a^

Compound (10 μM) ^b^	Inhibition (%)
**3**	88.36 ± 10.93
**6**	82.11 ± 9.31
**7**	88.36 ± 10.93
**8**	77.78 ± 4.24
**9**	77.73 ± 4.85
**11**	92.42 ± 9.50
Control	100.00 ± 9.90
Untreated Control	41.91 ± 0.04

^a^ Inhibition of osteoclast differentiation was reflected in the reduction of TRAP activity. TRAP-positive multinucleated osteoclasts (control, obtained from RANKL-induced RAW 264.7 cells) served as a positive control, while untreated cells (untreated control, without RANKL induction) served as a negative control. Values are expressed as a percentage of the control (mean ± standard deviation, *n* = 3). ^b^ Compounds **1**–**2**, **4**–**5**, and **10** showed no inhibitory effects on TRAP activity at 10 μM.

## Supplementary Materials

The following are available online. 1D/2D-NMR, CD, and HR-ESI-MS spectra of compounds **1** and **2**.
