# Supplementary material for: Antioxidant and Anti-Osteoporosis Activities of Chemical Constituents of the Stems of Zanthoxylum piperitum
Source: Molecules. 2018 Feb 18;23(2):457. doi: 10.3390/molecules23020457 (PMC6017793; doi:10.3390/molecules23020457)
Supplement: Supplementary file 1 [file molecules-23-00457-s001.pdf]

Supplementary Materials: Antioxidant and anti-osteoporosis activities of chemical constituents of the stems of *Zanthoxylum piperitum*

Seo Young Yang, Sang-Hyun Lee, Bui Huu Tai, Hae-Dong Jang and Young Ho Kim

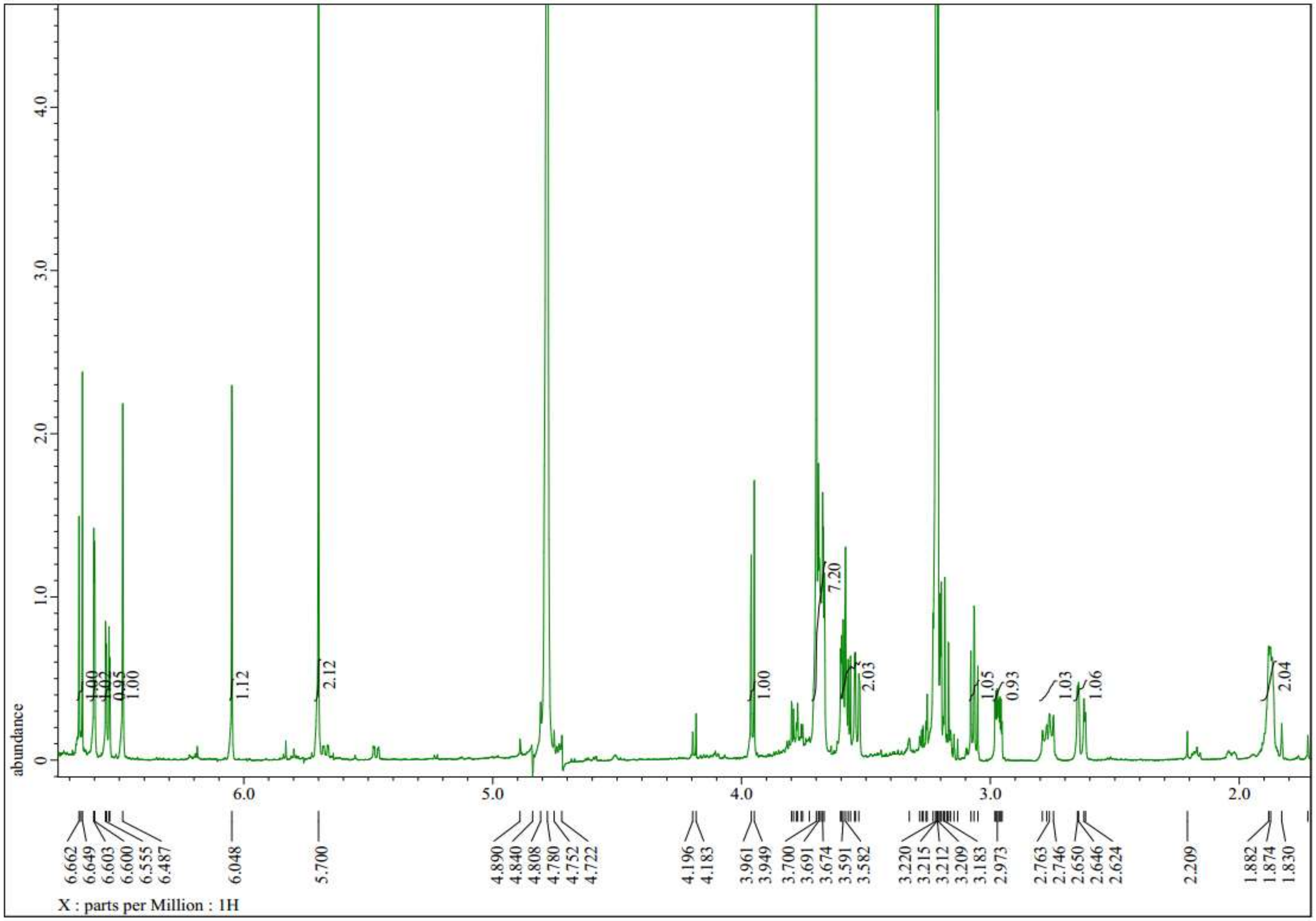

Figure S1. ^{1}H-NMR spectrum of compound **1** (600 MHz, CD_3OD).

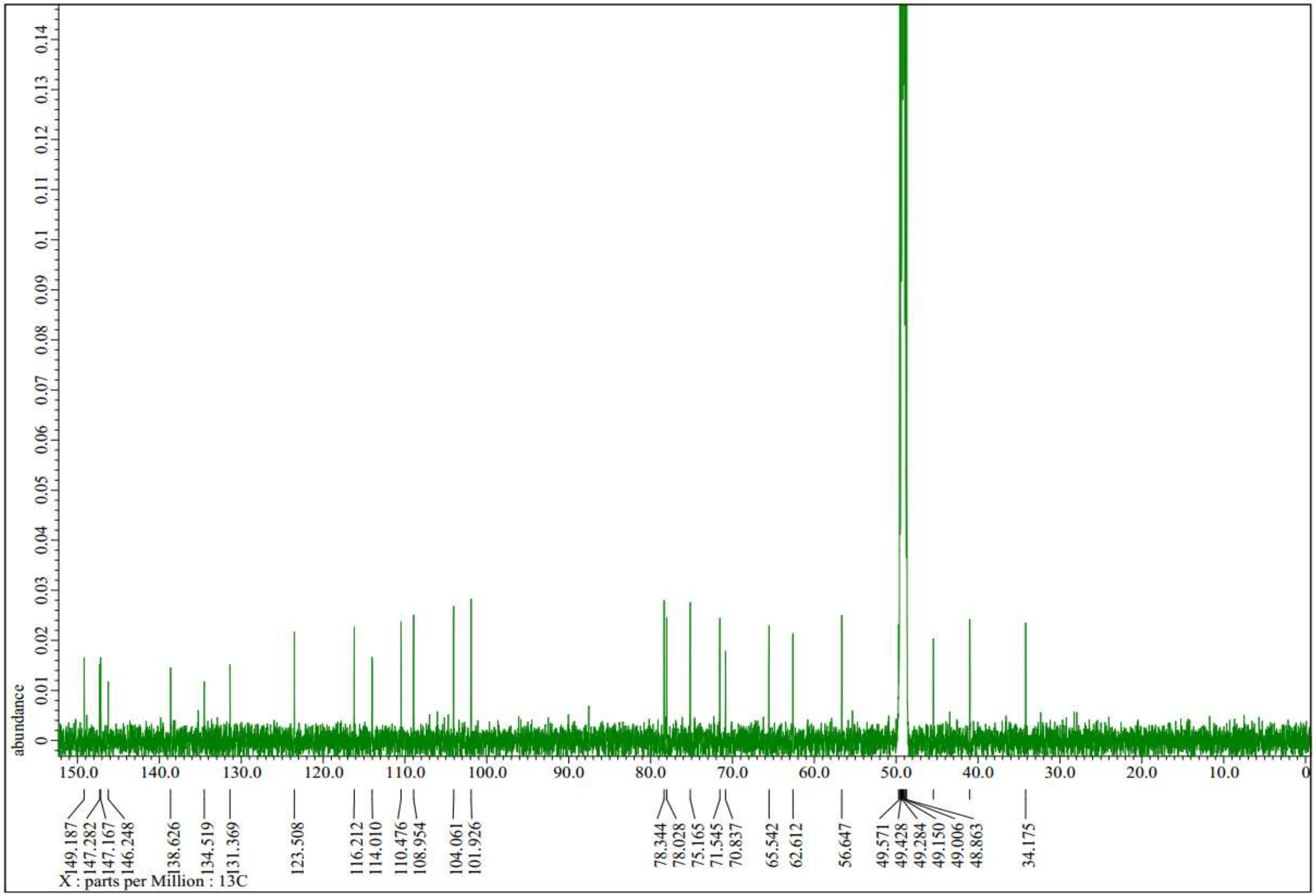

Figure S2. ^{13}C-NMR spectrum of compound **1** (150 MHz, CD_3OD).

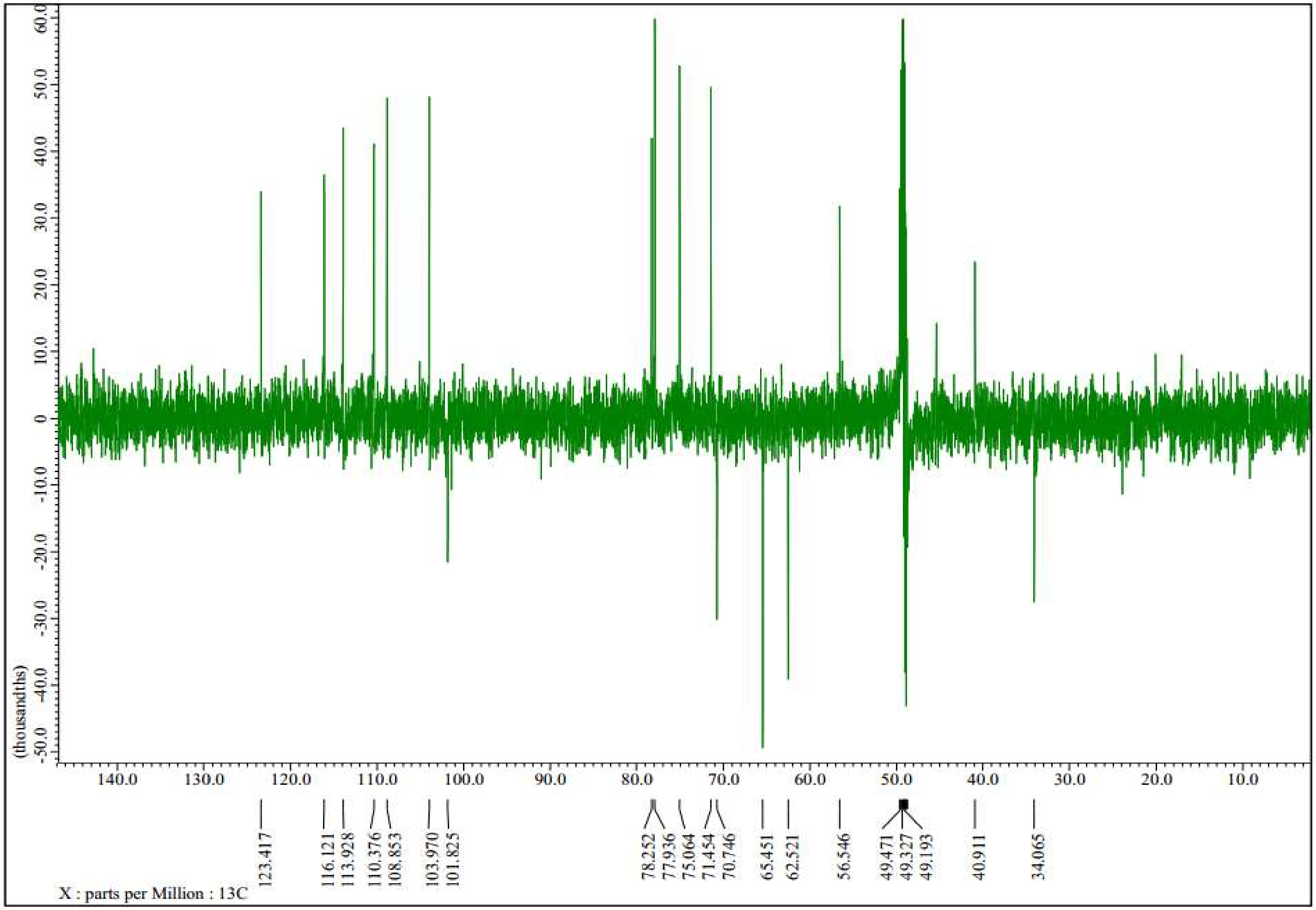

Figure S3. DEPT-135 spectrum of compound **1** (CD_3OD).

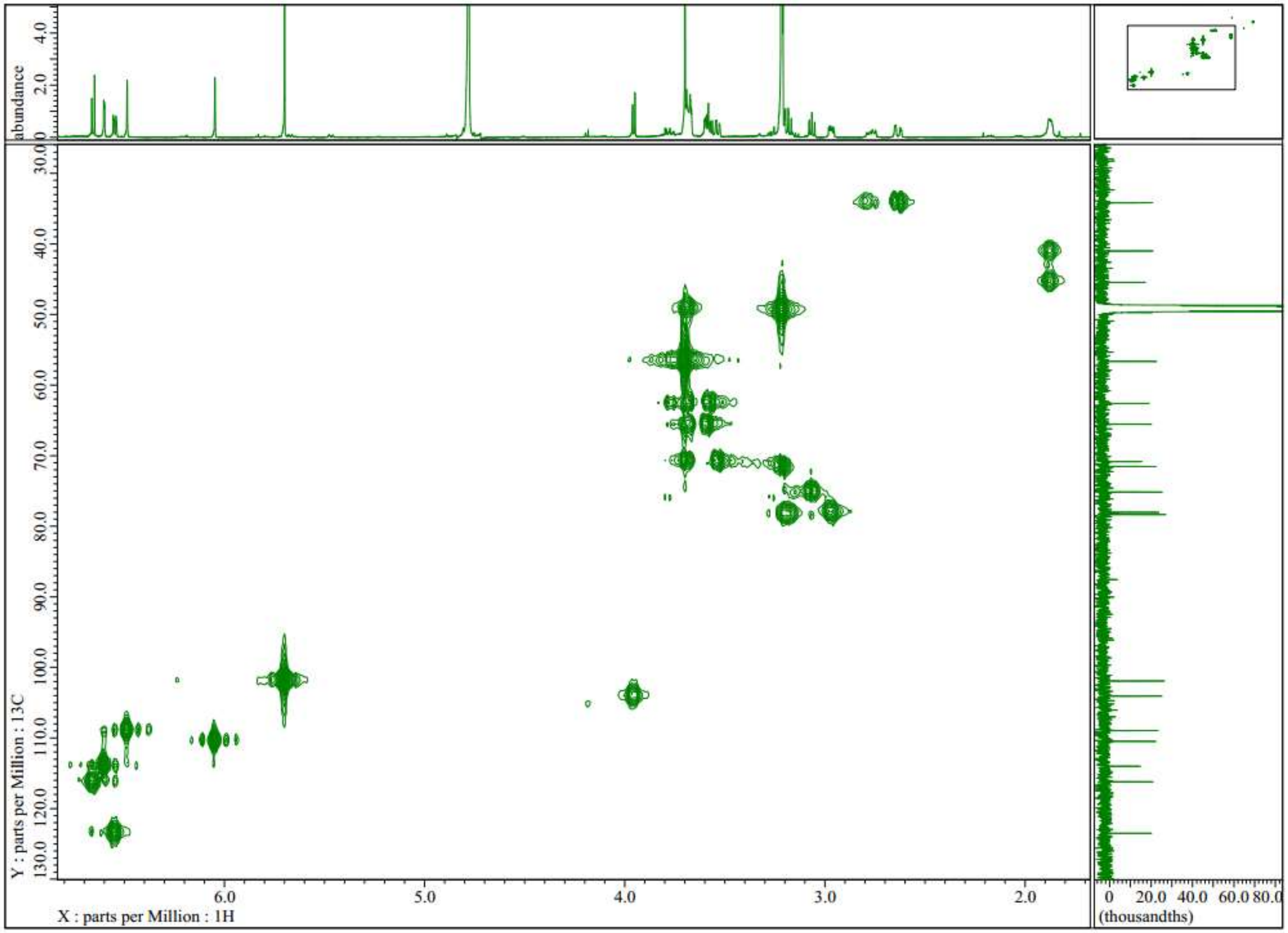

Figure S4. HMQC spectrum of compound **1** (CD_3OD).

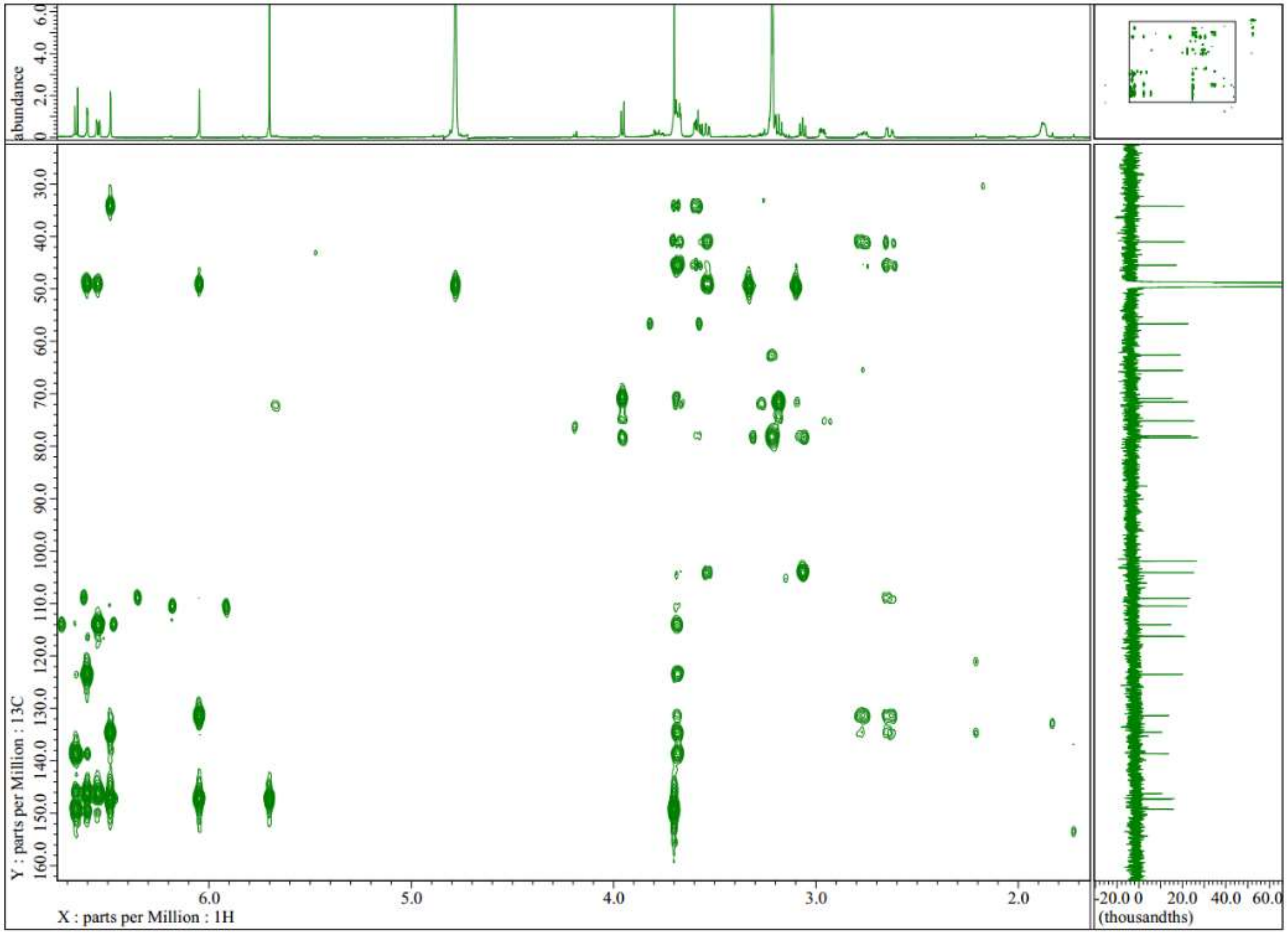

Figure S5. HMBC spectrum of compound **1** (CD_3OD).

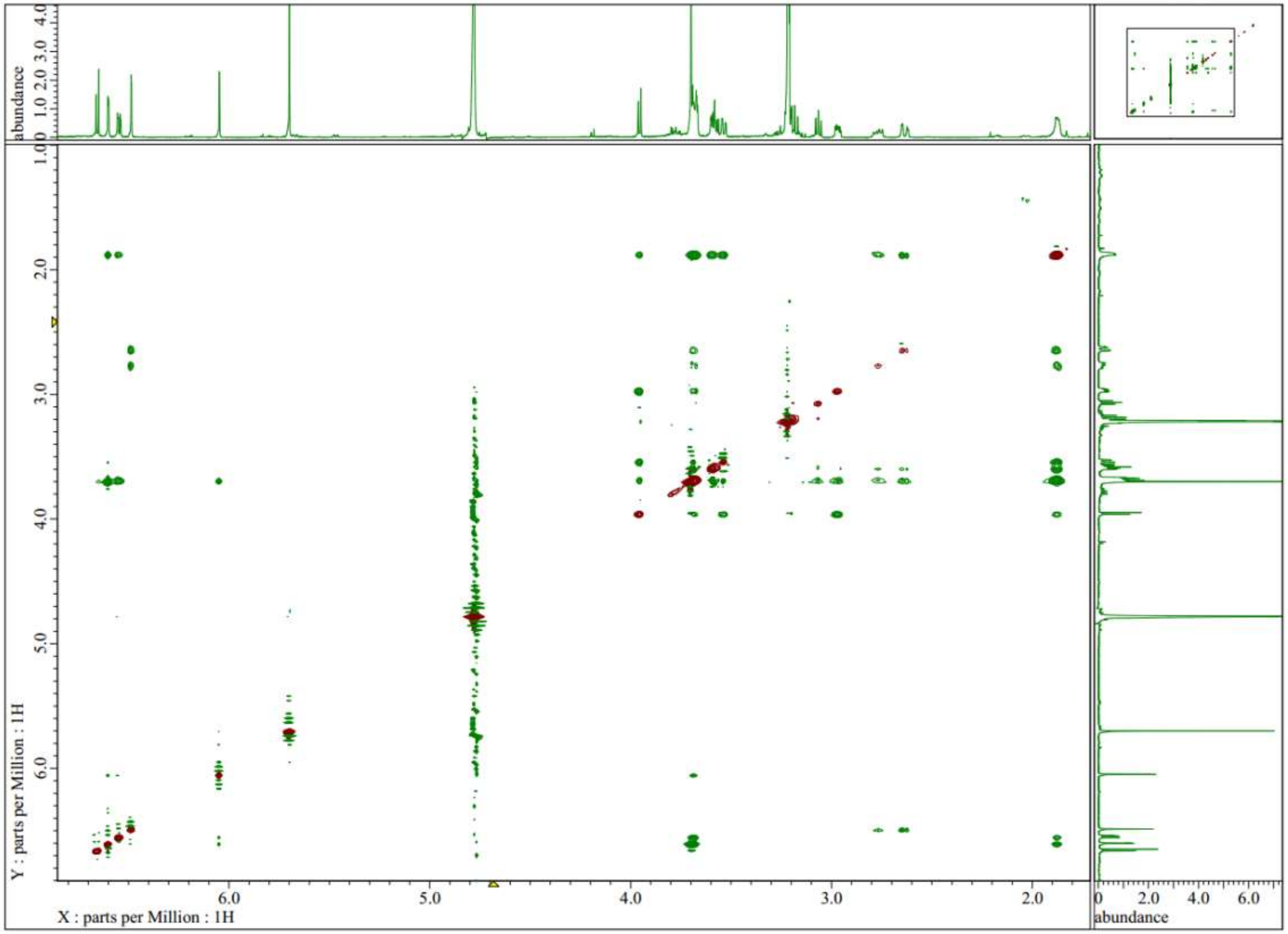

Figure S6. NOESY spectrum of compound **1** (CD_3OD).

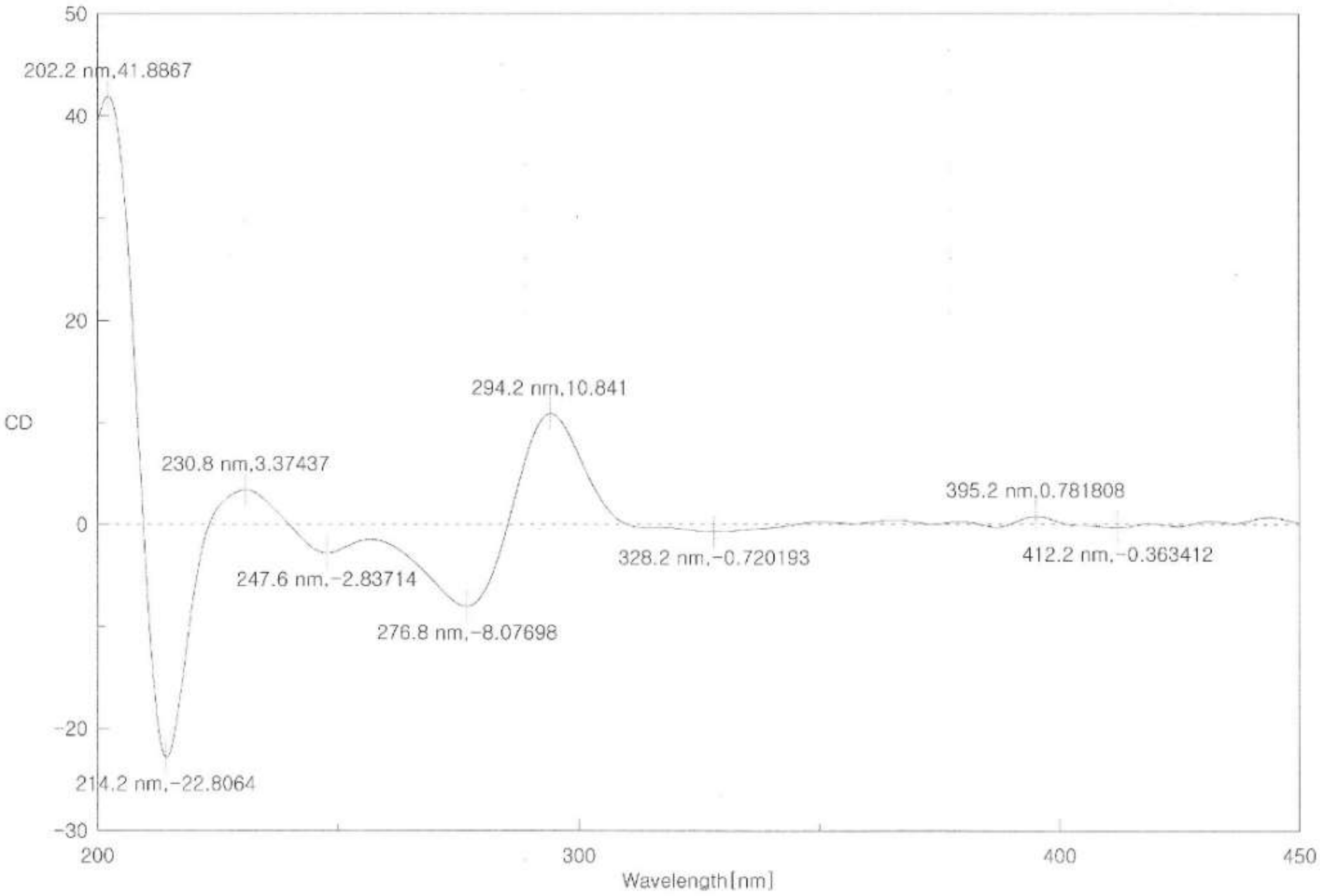

Figure S7. CD spectrum of compound **1**.

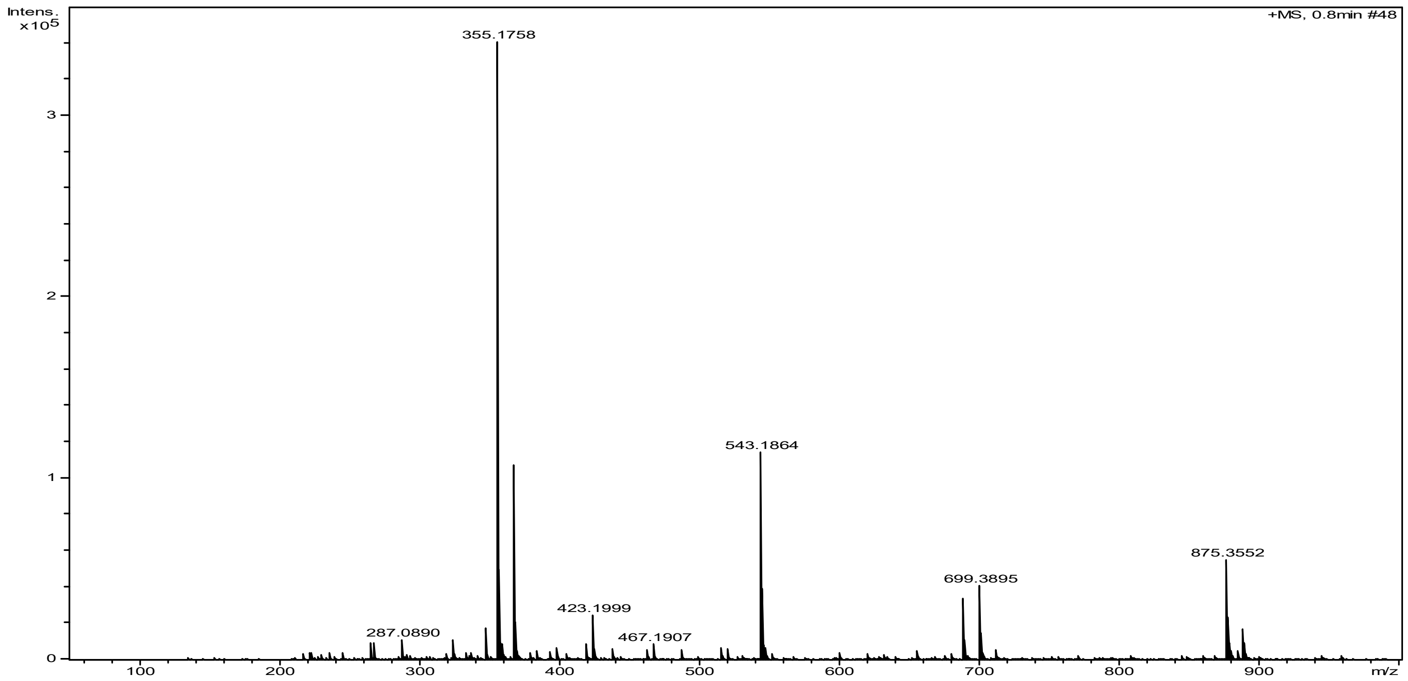

Figure S8. HR-ESI-MS spectrum of compound **1**.

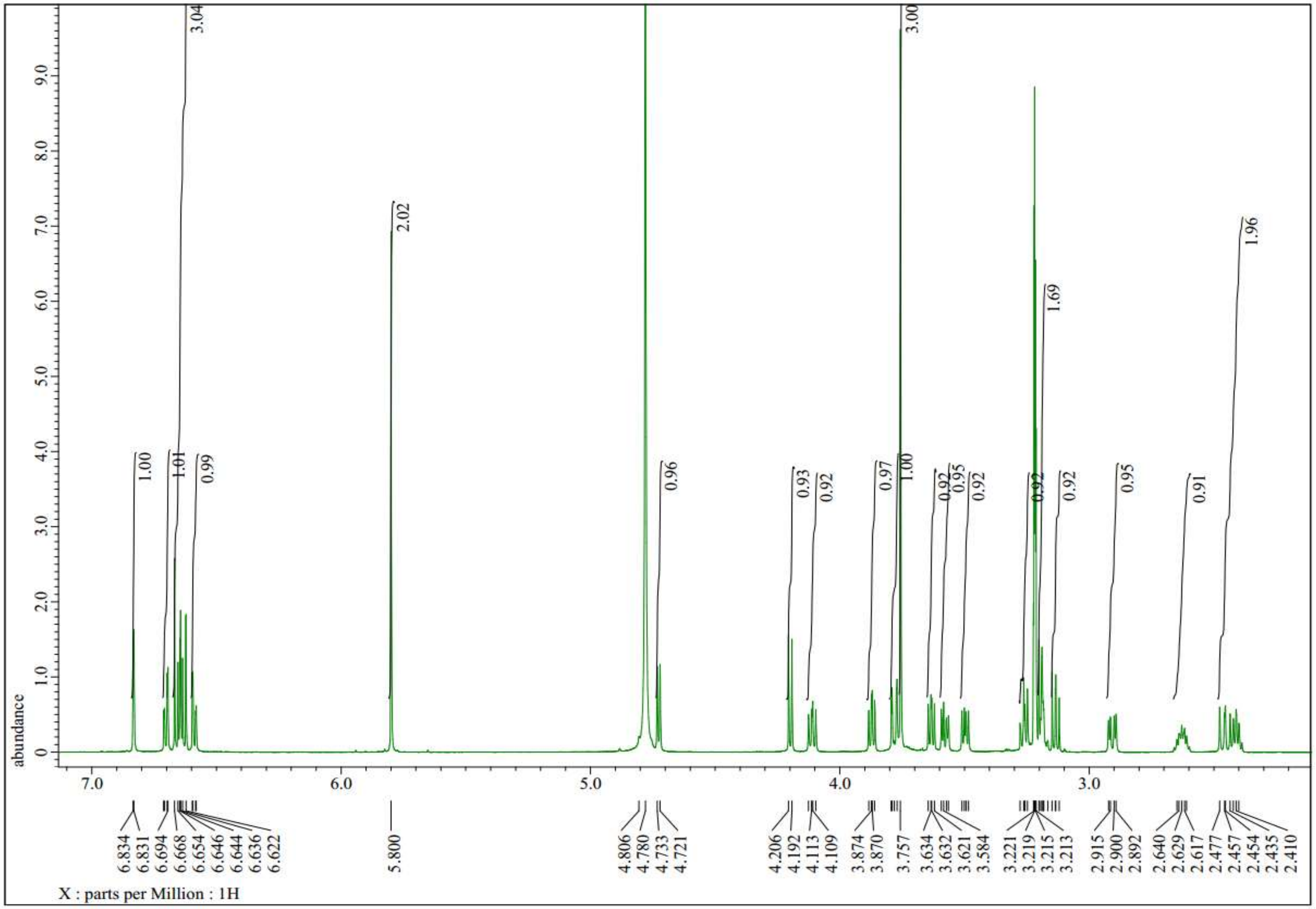

Figure S9. ^{1}H-NMR spectrum of compound **2** (600 MHz, CD_3OD).

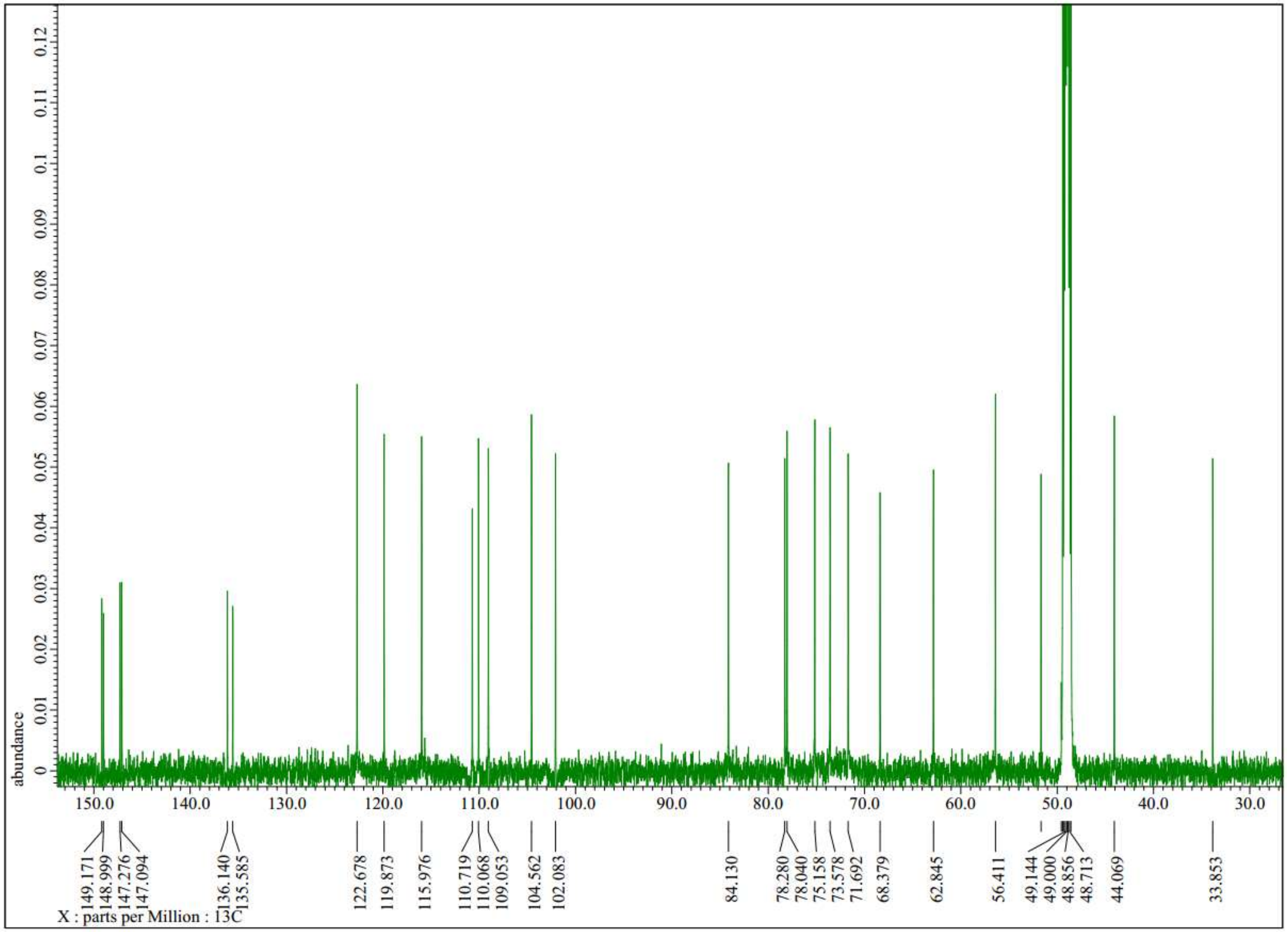

Figure S10. ^{13}C-NMR spectrum of compound **2** (150 MHz, CD_3OD).

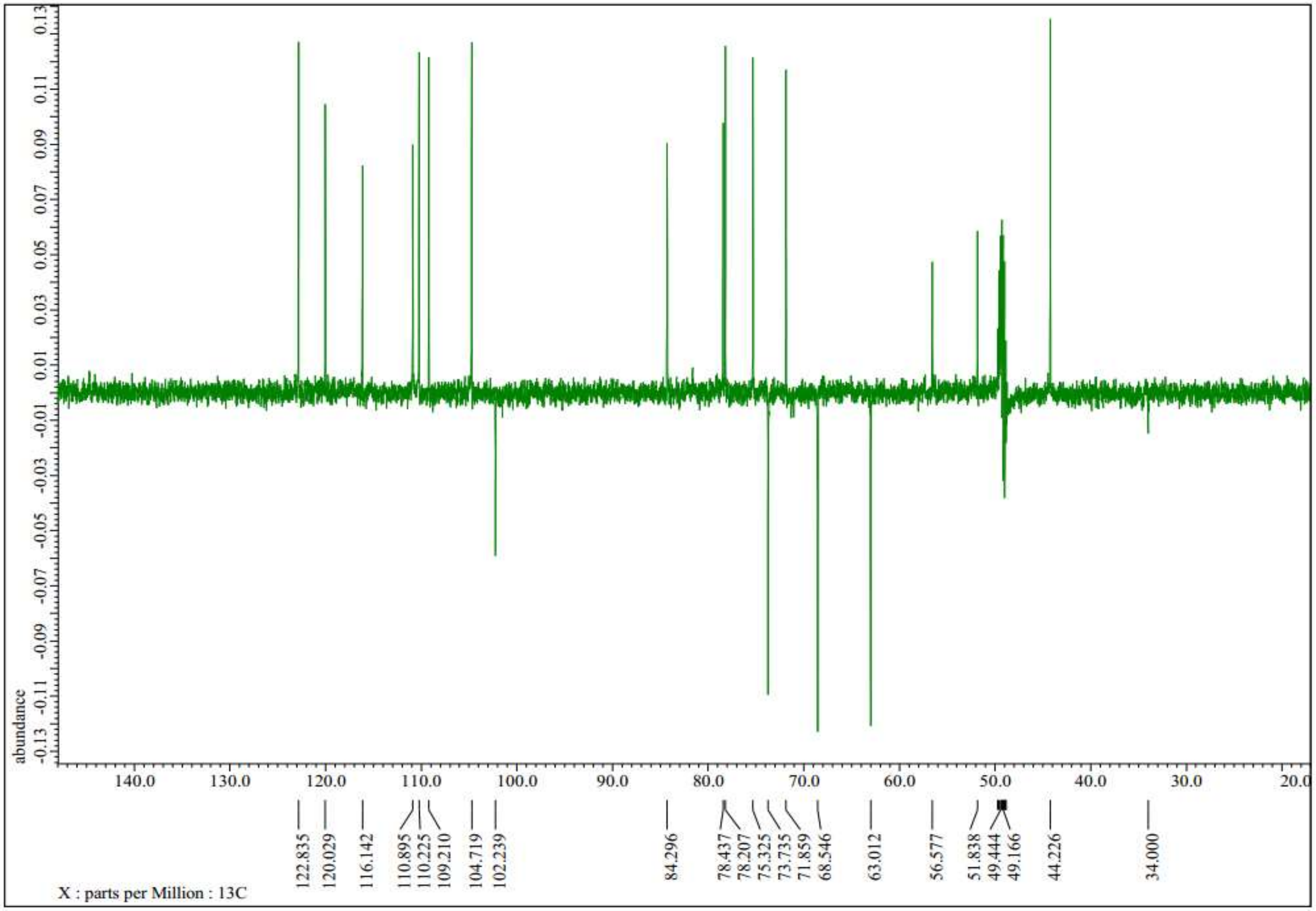

Figure S11. DEPT-135 spectrum of compound **2** (CD_3OD).

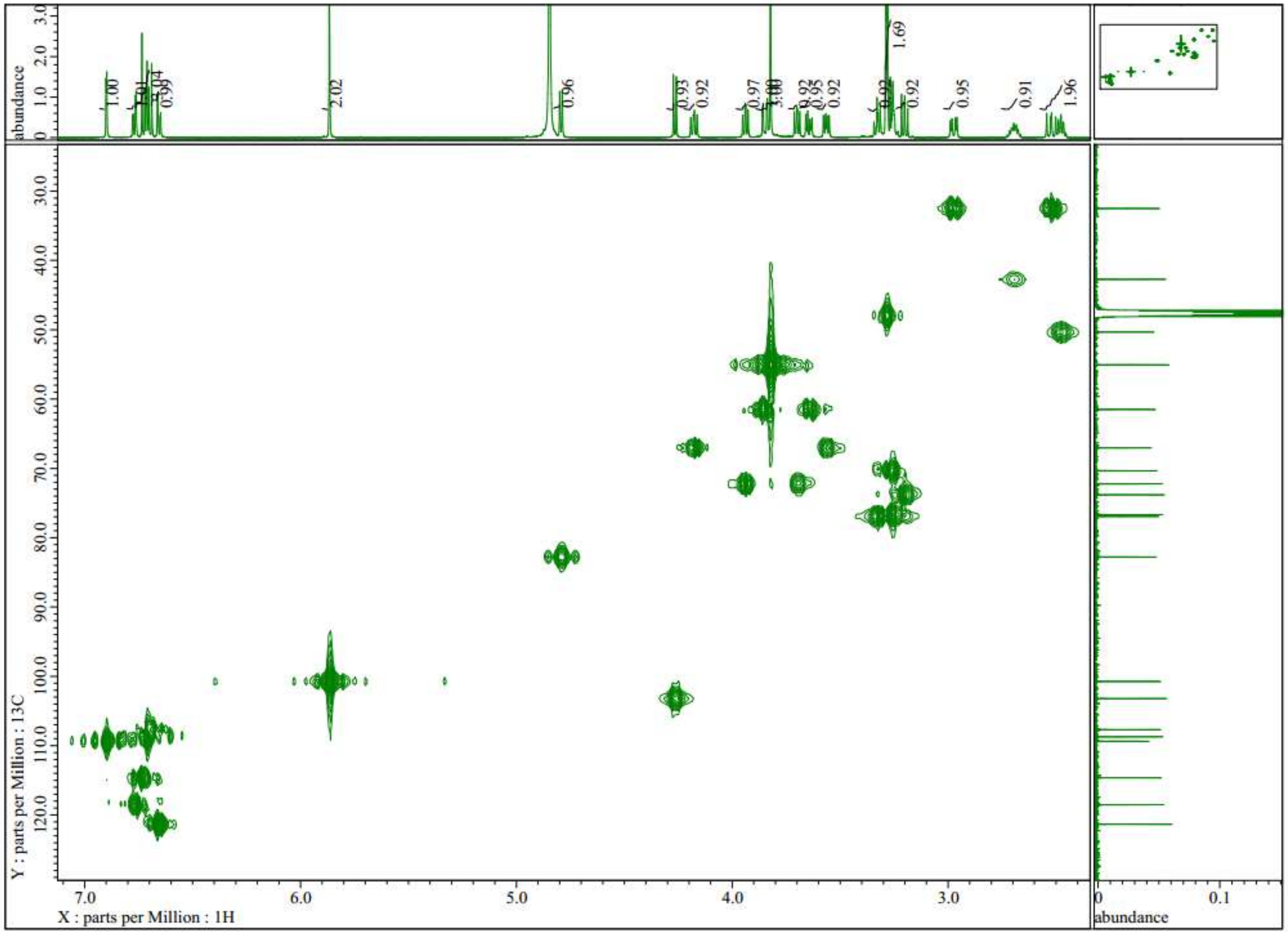

Figure S12. HMQC spectrum of compound **2** (CD_3OD).

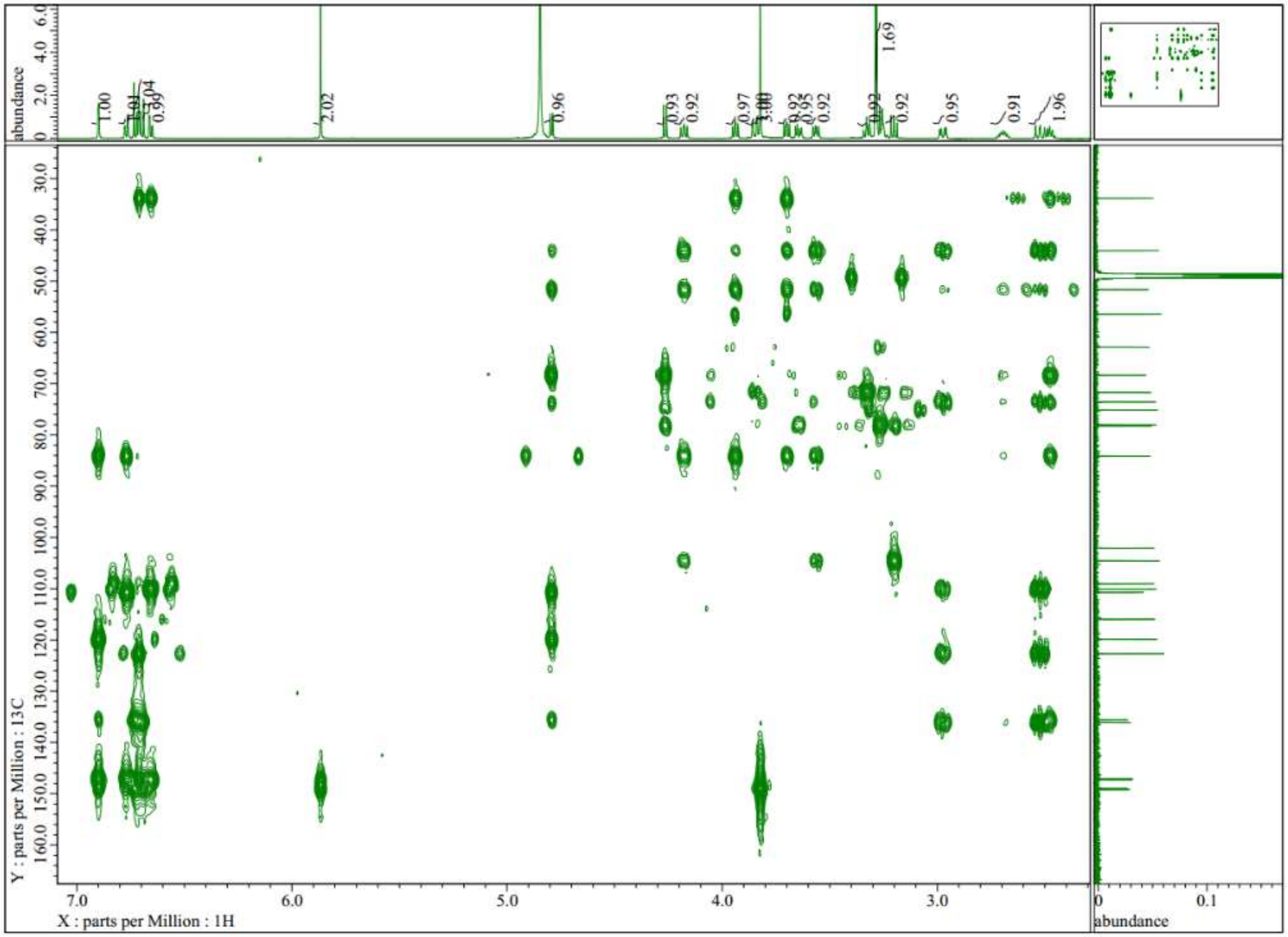

Figure S13. HMBC spectrum of compound **2** (CD_3OD).

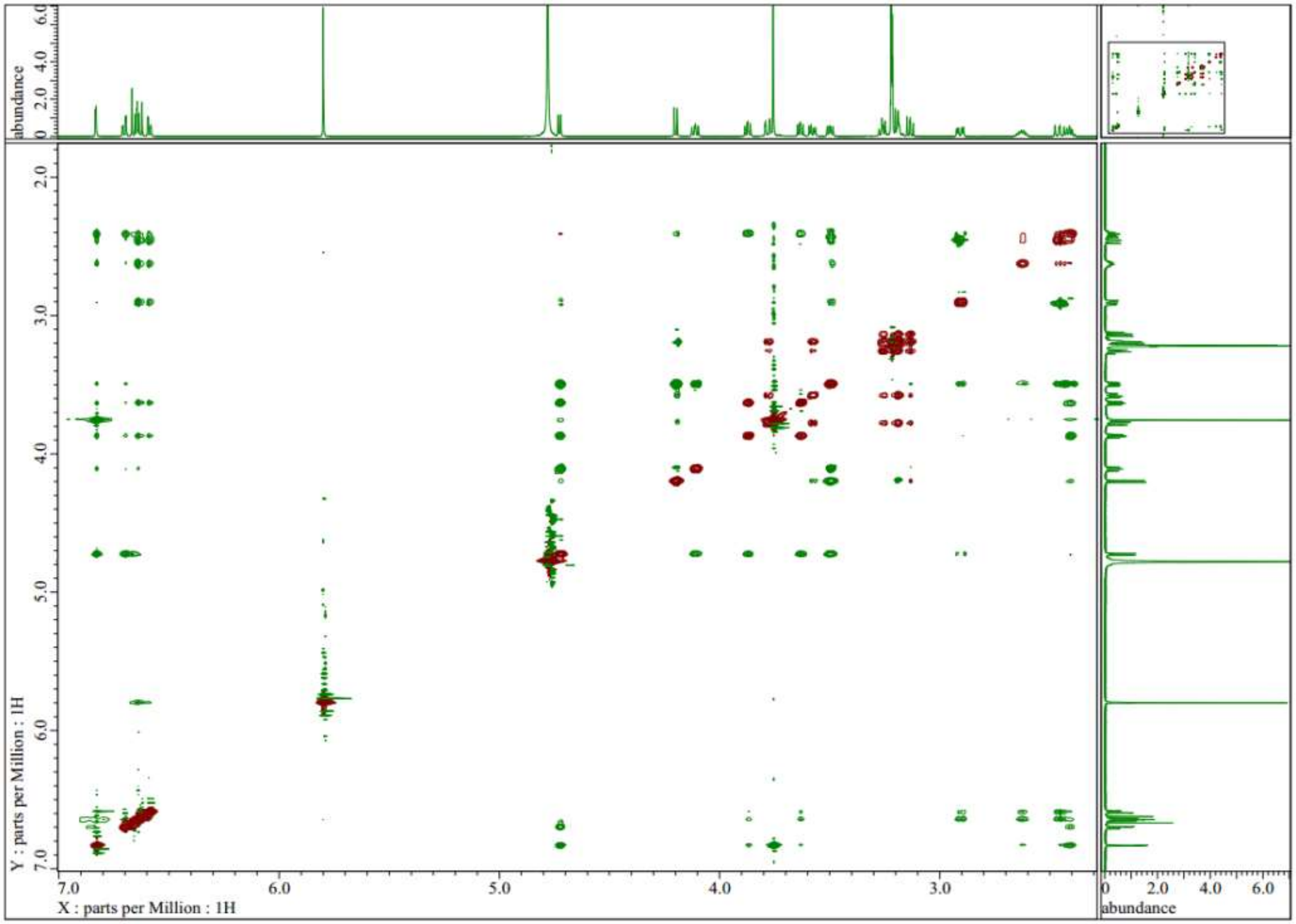

Figure S14. NOESY spectrum of compound **2** (CD_3OD).

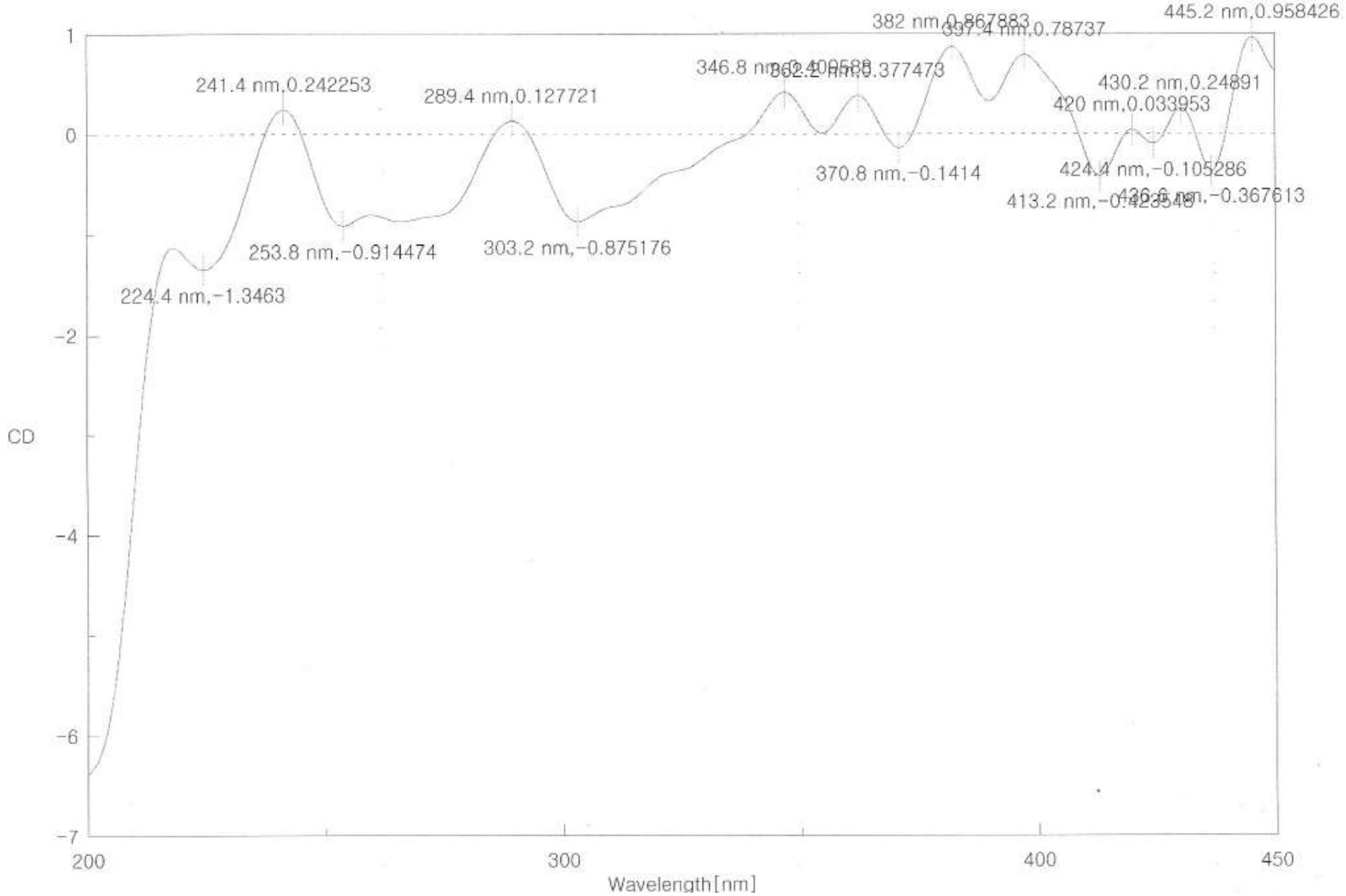

Figure S15. CD spectrum of compound **2**.

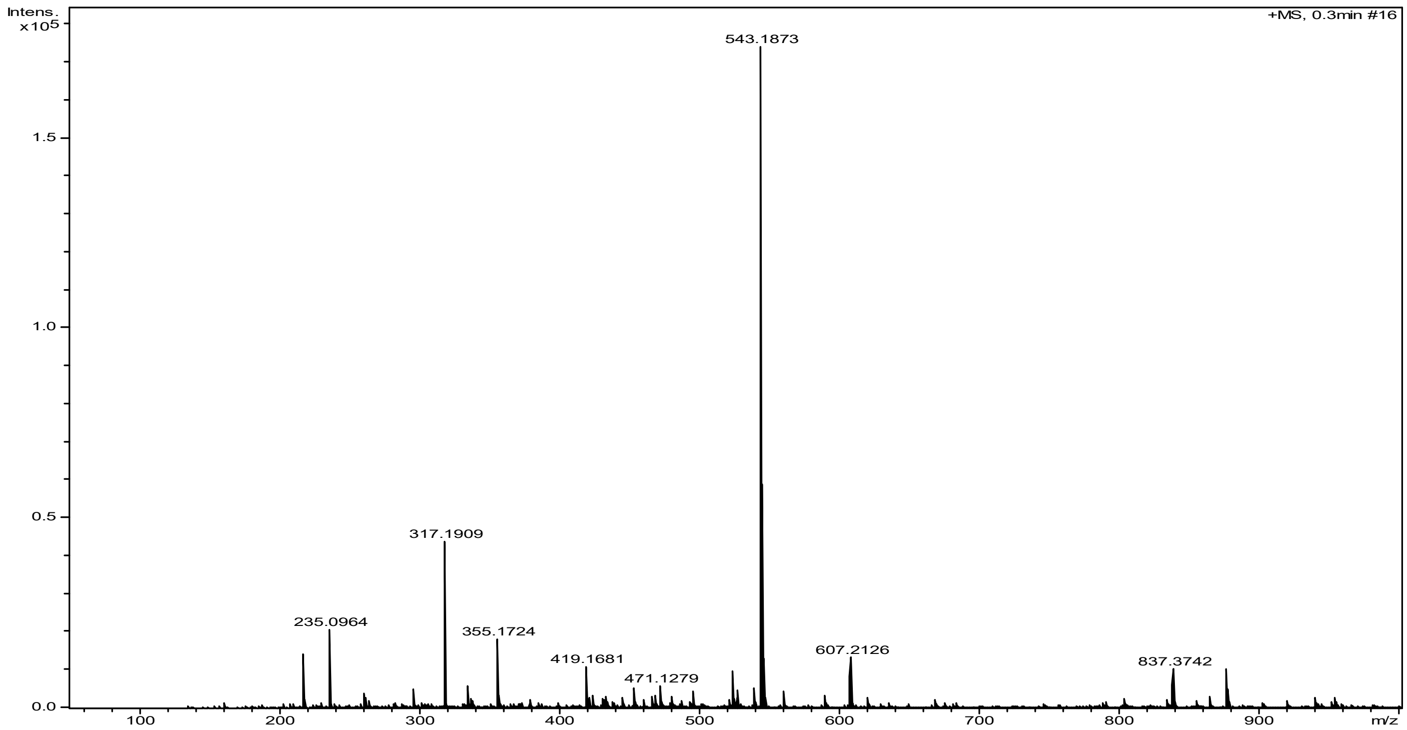

Figure S16. HR-ESI-MS spectrum of compound **2**.
